# Developing an Improved Survival Prediction Model for Disease Prognosis

**DOI:** 10.3390/biom12121751

**Published:** 2022-11-25

**Authors:** Zhanbo Chen, Qiufeng Wei

**Affiliations:** China-ASEAN Institutes of Statistics & Guangxi Key Laboratory of Big Data in Finance and Economics, Guangxi University of Finance and Economics, Nanning 530003, China

**Keywords:** survival prediction, machine learning, deep forest, self-supervised learning

## Abstract

Machine learning has become an important research field in genetics and molecular biology. Survival analysis using machine learning can provide an important computed-aid clinical research scheme for evaluating tumor treatment options. However, the genomic features are high-dimensional, which limits the prediction performance of the survival learning model. Therefore, in this paper, we propose an improved survival prediction model using a deep forest and self-supervised learning. It uses a deep survival forest to perform adaptive learning of high-dimensional genomic data and ensure robustness. In addition, self-supervised learning, as a semi-supervised learning style, is designed to utilize unlabeled samples to improve model performance. Based on four cancer datasets from The Cancer Genome Atlas (TCGA), the experimental results show that our proposed method outperforms four advanced survival analysis methods in terms of the C-index and brier score. The developed prediction model will help doctors rethink patient characteristics’ relevance to survival time and personalize treatment decisions.

## 1. Introduction

In the last two decades, bioinformatics technology has obtained rapid development to provide efficient computer-aid ways to diagnose diseases, and bioinformatics with machine learning can make significant breakthroughs in the tumor diagnosis [1]. The rapid development of high-throughput sequencing technology has demonstrated that gene expression profiling may be used to predict various clinical phenotypes [2]. A survival prediction model has been used to analyze and grasp the relationships between medical characteristics and survival time of patients in recent years [3]. Cancer prognosis was assessed by the survival analysis method to provide valuable information [4]. As usual, high-dimensional candidate genomic features severely reduced the performance of treatments of various predicted clinical phenotypes [5,6]. There is a key challenge to improving the prognostic accuracy in survival prediction models. The cox proportional hazard (CPH) model, commonly known as the cox model, is widely used in survival analysis tasks [7]. It can predict a risk score according to the characteristics or covariates of a set of patient data and correct censored data effectively. Even if the cox model as a linear model has many advantages, a disadvantage of it is that it cannot express the complex nonlinear relationship between the logarithmic risk ratio and static covariates [8].

Therefore, a machine learning-based CPH model was utilized to solve a complex nonlinear survival analysis problem [9]. Support vector machine (SVM) is a classical machine learning approach to process high-dimensional features by incorporating ranking and regression constraints [10]. Thus, an SVM-based CPH method can enhance the learning of high-dimensional data, whereas the hazard was not directly incorporated into data in the model. Deep learning networks are used to determine gene expression data that predict cox regression survival in breast cancer [11]. A broad analysis was performed on TCGA cancers using a variety of deep learning-based models applied to the survival prognosis of cancer patients [12]. The random forest is an ensemble learning method that can find the mating survival rate of each patient accurately. Therefore, a random survival forest methodology was investigated through the extended the random forest method, which can analyze the right-censored survival data [13].

A deep forest (DF) model is a decision-tree-based ensemble learning method including a deep nonneural network type, which has good performance in many tasks [14]. Additionally, deep forests have developed two types, namely, random forests and completely random-tree forests, which can help to improve diversity of the learning model. A deep survival forest based on deep forest was proposed to construct a model and replace the original random forest with the corresponding survival analysis model. As a tracking algorithm implemented in a deep survival forest and elastic network cox cascade, it can be regarded as a link between deep forest levels [15].

Any dataset will contain a large number of unlabeled samples because genome-wide gene expression profiling is still too expensive to be used with academic laboratories to research the rich gene expression analysis method [16]. Thus, in order to improve the model’s learning ability, semi-supervised learning (an incremental learning technique) is investigated to obtain more labeled data from unlabeled samples. Self-supervised learning, an intuitive pseudo-labeling SSL technique, is a general learning framework that relies on a prelearning task formulated by unsupervised labeled data. In this study, we employed self-supervised learning techniques that are designed to learn a useful global model from labeled data. Many recent self-supervised methods have received increasing attention to solve the dilemma of a lack of labels. For example, a twin self-supervision–semi-supervised learning approach is presented to embed self-supervised strategies into a semi-supervised framework to simultaneously learn from few-shot-labeled images and vast unlabeled images [17]. Liu et al. [18] proposed a self-supervised mean-teacher method for semi-supervised learning which combines the pre-training of self-supervised mean with semi-supervised fine-tuning to improve the representativeness of the mean-teacher. To tackle these problems, Song et al. [19] proposed a self-supervised semi-supervised learning framework to tackle the problem of sparsely labeled hyperspectral image recognition.

Motivated by the lack of relevant research, we attempted to exploit the deep survival forest with self-supervised learning in survival analysis tasks. Recently, several survival analysis methods with genomic feature selection have been investigated to predict the survival time of patients precisely. This has become a key technique to improve performance in learning models [20]. For example, a deep forest model based on feature selection is proposed to reduce the redundancy of features, and could be adaptively incorporated with the classification model [21]. Zhu et al. [22] presented an ensemble feature-selection–deep-forest method which outperformed the traditional machine-learning methods. In the prediction of protein–protein interactions, elastic net deep forest is utilized to optimize the initial feature vectors and boost the predictive performance [23]. Stable feature selection can efficiently avoid negative influences from added or removed training samples [24]. Thus, we identified disease-causing genes by investigating stable LASSO regularization in survival analysis. In this paper, we propose a self-supervised method using a deep forest algorithm to improve survival prediction performance—deep forest can learn from high-dimensional genome data efficiently; and semi-supervised learning such as self-supervised learning provides more labeled samples to train a global model.

Though extensive testing on the real-world TCGA cancer datasets, the results show that the proposed DFSC method has high prediction accuracy even if high-dimensional survival data are used. The rest of this article is organized as follows. Section 2 describes our method and experimental dataset. The results are displayed and discussed in Section 3. Finally, conclusions are presented in Section 4.

## 2. Methods

### 2.1. Deep Survival Forest

The training dataset *D* usually including *n* triplets ${\left(x,\sigma_{i},T_{i}\right)}_{i=1}^{n}$, where x is the vector of the patient characteristics, *T* is time-to-event of the patients, i.e., patients’ diagnosis time interval from the start time to the event time occurs. σ=1 corresponds to an uncensored observation, and σ=0 indicates a censored observation. The deep survival forest learning goal is to estimate the time to the event *T* for a new patient using a feature vector x. Thus, the deep survival forest H(x) is defined as the integral of hazard function h(t); it is given a rate of events at time *t* to show that no event happened before time *t*. The hazard function h(t|x) at time *t* given the training data x is defined as follows:

(1) $$h\left(t|x\right)=h_{0}\left(t\right)e^{g\left(x\right)b^{T}},$$

where $h_{0}\left(t\right)$ is a baseline hazard function, *b* is regression coefficients, and g(x) is deep forest model. To obtain parameters of the learning model, the partial likelihood is used in the form:

(2) $$\ell \left(b\right)=\prod_{j=1}^{n}{\left[\frac{e^{g(x_{j}b^{T})}}{\sum_{i\in R_{j}}e^{g(x_{i}b^{T})}}\right]}^{\sigma_{j}}$$

**Figure 1:**
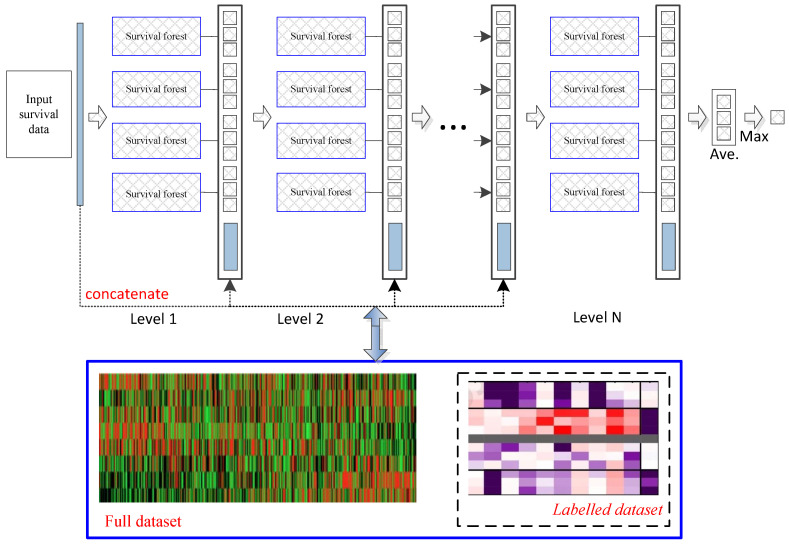
Flowchart of the learning method. Each level of the cascade consists of deep survival forests, which directly learn cancer prognosis prediction with multiple decision trees. All connected features will be optimized to obtain a more compact feature set and then transferred to the next level. Our model can use both labeled and unlabeled datasets. Deep survival forest can labels unlabeled data to augmented training datasets.

Deep survival forest provides an alternative method for deep survival neural networks to learn the multilevel structure representations with fewer hyperparameters. Figure 1 describes a brief deep survival forest procedure which directly learns cancer prognosis prediction with multiple decision trees, rather than learning through the hidden layers of neural networks. In addition, due to the strong learning ability of random forests, an ensemble of forests can achieve more accurate cancer prognosis prediction. In this work, we used the original parameters to iteratively perform the deep survival forest process in the experiments [15,25]. The convergence condition is that the training samples (the combination of the original training and the pseudolabeled samples) obtain the optimal solution by using the pseudolabeled samples ${\left(x\right)}_{i}^{n}$. In particular, a deep survival forest with labeled samples was utilized to train a model to label unlabeled samples. Then, combining labeled and pseudolabeled samples can achieve higher performances. Deep survival forest functions are similar to those of the random forest ensemble model.

### 2.2. Self-Supervised Learning via Unlabeled Examples

To further improve the model, we leverage the unlabeled data. We use a pre-learning deep survival forest as a teacher model to improve labels for training a student network. The unlabeled samples distillation loss is minimized as follows:

(3) $$\ell^{distill}=\sum_{x_{i}\in D}\left[\sum_{y}P^{T}\left(y|x_{i};\tau \right)logP^{S}\left(y|x_{i};\tau \right)\right]$$

where $P\left(y|x_{i}\right)=exp\left(f\left(x_{i}\right)\left[y\right]/\tau \right)/\sum_{y^{\prime }}exp\left(f\left(x_{i}\right)\left[y^{\prime }\right]/\tau \right)$, and τ is a scalar temperature parameter. The teacher model, which produces $P^{T}\left(y|x_{i}\right)$, is fixed during the distillation; only the student model, which produces $P^{S}\left(y|x_{i}\right)$, is trained.

### 2.3. TCGA Gene Expression Data

In this study, four gene expression datasets from non-currently embargoed TCGA projects were obtained from the TCGA data portal (https://portal.gdc.cancer.gov/, accessed on 20 June 2022). Only GEP obtained using Illumina HiSeq 2000 were retrieved. Table 1 describes aspects of the experimental datasets.

## 3. Results

### 3.1. Experimental Brief

To test model robustness, fivefold cross-validation was used to estimate the different survival prediction algorithms; i.e., the dataset was divided into five folds of approximately equal sizes. Next, each fold was used as a test separately, and other data were utilized as the training dataset. Additionally, four SOTA methods were used to evaluate the performance of our method: LASSO-COX, survival SVM (support vector machine), RSF (random survival forest), and EXSA (survival analysis of gradient boosting). In the comparison of survival analysis, the concordance index (CI or C-index) and brier score, key metrics of the survival prediction model, were used to evaluate the performance [26]. If the predicted survival time of a patient with a longer life span is larger, the prediction of the patient is considered to be consistent with the outcomes. The C-index is a generalization of the area under the ROC curve (AUC) that can take into account censored data. The C-index can predict the data (consist of right-censored data) to measure the overall the survival model prediction performance, which ranges in an interval [0,1]. The higher the value of C-index, the higher the predictive accuracy of the survival prediction model. Brier score represents the average squared distances between the observed survival status and the predicted survival probability, which is influenced by both discrimination and calibration simultaneously. The two main components decomposed from brier score are reliability and resolution, which, respectively, measure the closeness between the predictive probabilities and true probabilities and the difference between the conditional probability and the predicted average value [27]. The brier score range is [0,1], and smaller values reflect excellent prediction performance.

In the setting of the experiment, portions of the four cancer datasets were treated as unlabeled samples to evaluate the learning performance of the proposed approach. Then, labeled and unlabeled samples were randomly selected in every iteration; furthermore, details about the dataset are shown in Table 2.

**Figure 2:**
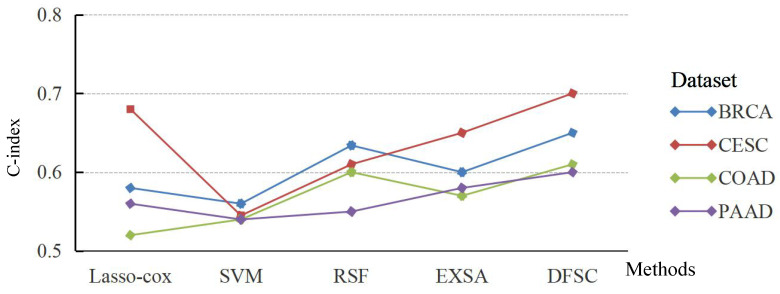
Average C-index achieved by learning model (higher is better) over multiple different train/test splits of each dataset.

**Figure 3:**
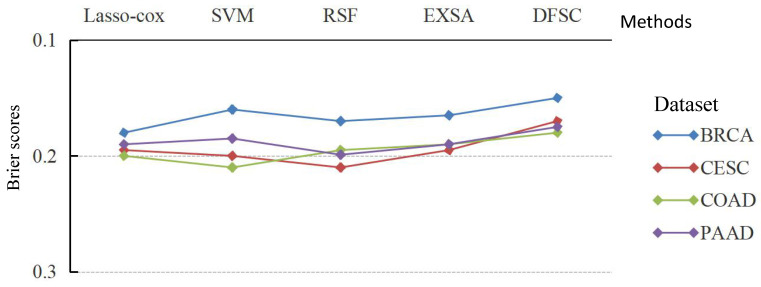
Average brier score achieved by learning model (lower is better) over multiple different train/test splits of each dataset.

The averaged C-index and brier score were used to display the performances of various survival prediction models on each experimental dataset by fivefold cross-validation. The average results of C-index and brier score are shown in Figure 2 and Figure 3 over multiple different test data.

Compared with the baseline algorithms Lasso–Cox, SVM, RSF, and EXSA, the average C-index of DFSC was 5.7% lower. DFSC outperformed EXSA, which is based on XGBoost; its average C-index was higher by 4%. These results indicate that DFSC achieves comparable accuracy to other models when using deep forest to predict patient survival rates. Therefore, the application of ensemble random forest can enable DFSC to analyze high-dimensional genomic features and achieve optimal performance.

In addition, the brier scores of the four different methods on four cancer datasets verify the effectiveness of the proposed algorithm in Figure 3. Meanwhile, Figure 3 indicates that DFSC is superior to the other models and the average brier score is 0.168—2.25%, 2%, 2.47%, and 1.62% lower than Lasso–Cox, survival SVM, RSF, and EXSA, respectively. That is, the DFSC model is optimal compared to other survival models according to the average brier score.

### 3.2. Discussion

Stable LASSO, as a computer-aided learning approach, was used in this work to further illustrate the advantage and interpratable nature of our method [28]. The top-20 genes selected by stable LASSO in the various datasets are listed in Table 3, Table 4, Table 5 and Table 6. When the stability scores of these genes are close to 1; the selected genes are robust. Additionally, the p-values are less than 0.05 in Table 3, Table 4, Table 5 and Table 6, which indicates that selected these genes are significant. Many studies consider functional analysis for gene expression. For example, MAGED1 in Table 3 acts as a tumor antigen and putative regulator of p53 transcription, as a candidate marker of acquired tamoxifen resistance [29]. TRIP12 in Table 4 leads to increased RNF168 levels, repressed DNA damage repair (DDR), increased 53BP1 foci, and enhanced radioresponsiveness [30]. CBLN2 in Table 5 is a CBLN family member and has been found to stabilize synapses by acting as a trans-synaptic link, binding with beta-neurexins of granule neurons and delta 2 glutamate receptors of Purkinje cells in the cerebellum [31]. TNNI1 in Table 6 shows the highest overexpression in cancers, showing the functional relevance of overexpression for developing novel therapies and diagnostic markers [32].

**Figure 4:**
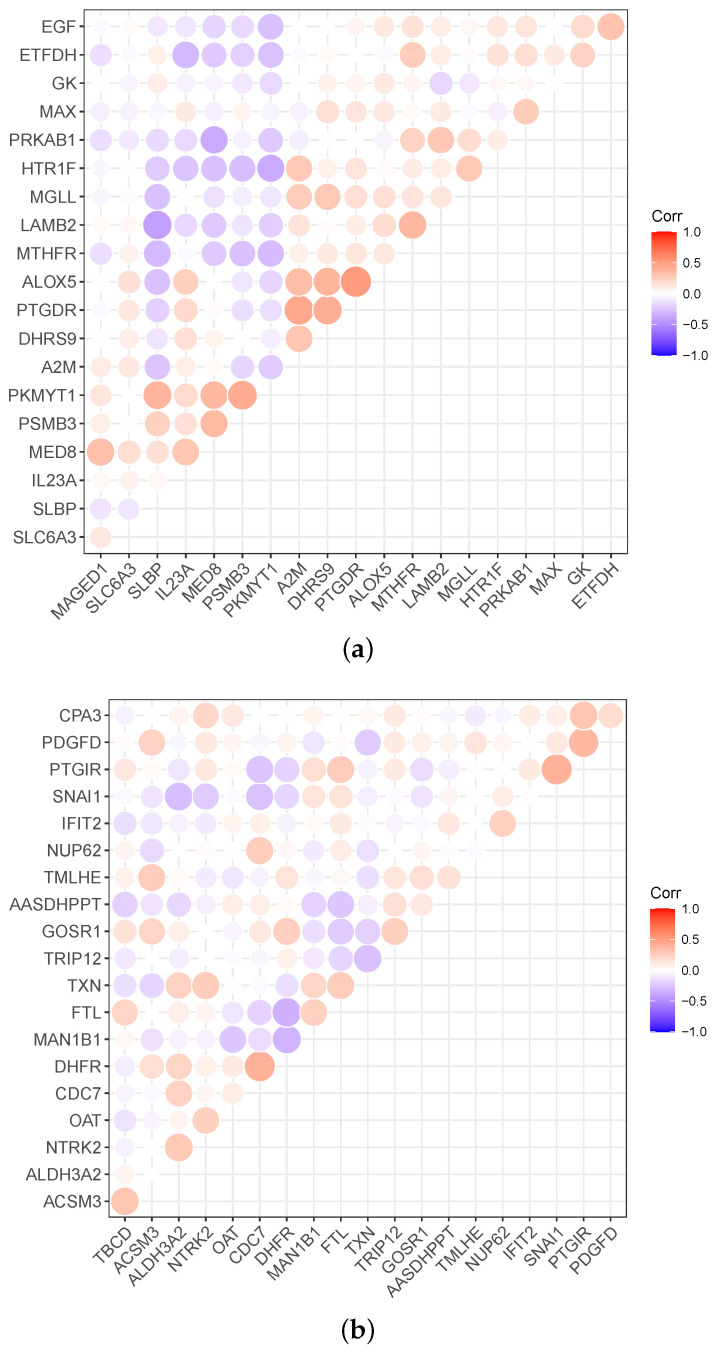

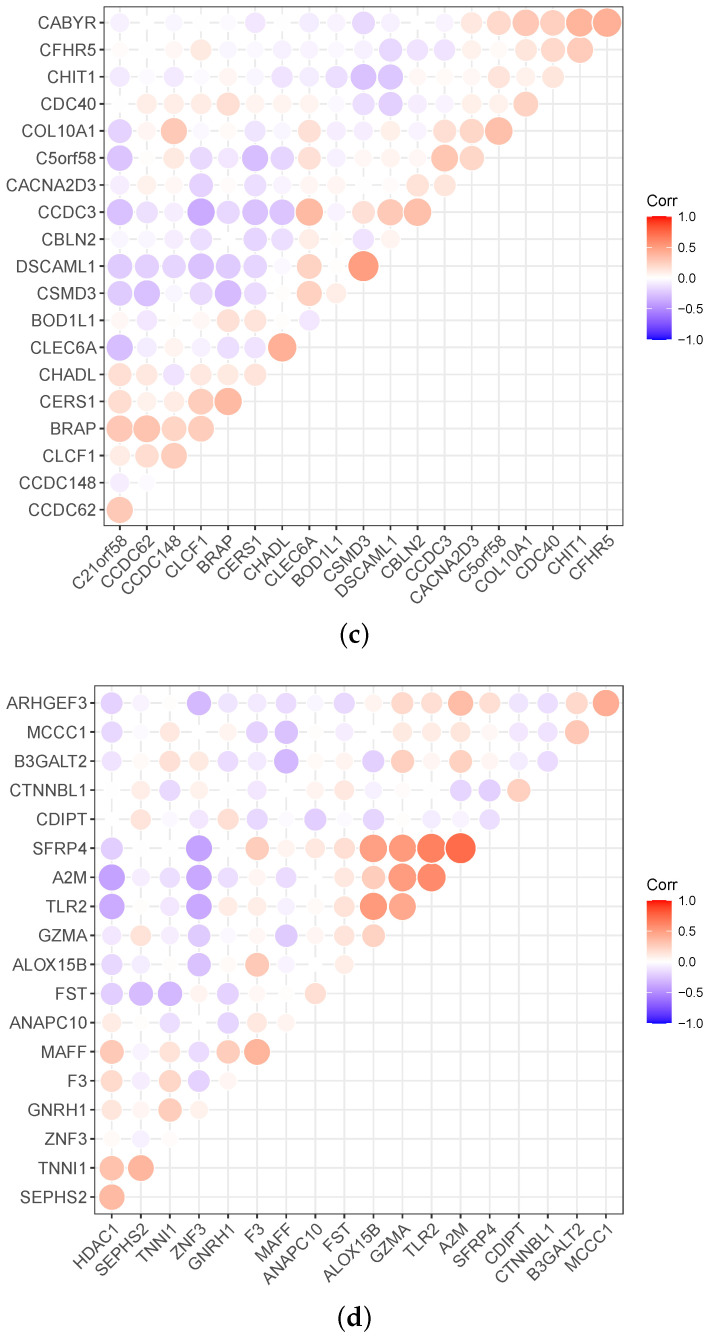
Relevance display via heat map for the four datasets: (**a**) BRCA (breast cancer), (**b**) CESC (cervical carcinoma cancer), (**c**) COAD (colorectal cancer), and (**d**) PAAD (pancreas cancer).

Meanwhile, the heatmap can expresses correlations between the genes, as shown in Figure 4. Red in Figure 4 indicates a positive correlation, and violet indicates a negative correlation. Correspondingly, the darker the color, the stronger the correlation. For example, the PKMYT1 is negatively correlated with the other 13 genes in breast cancer data, and MED8 is positively correlated with the other four genes.

**Figure 5:**
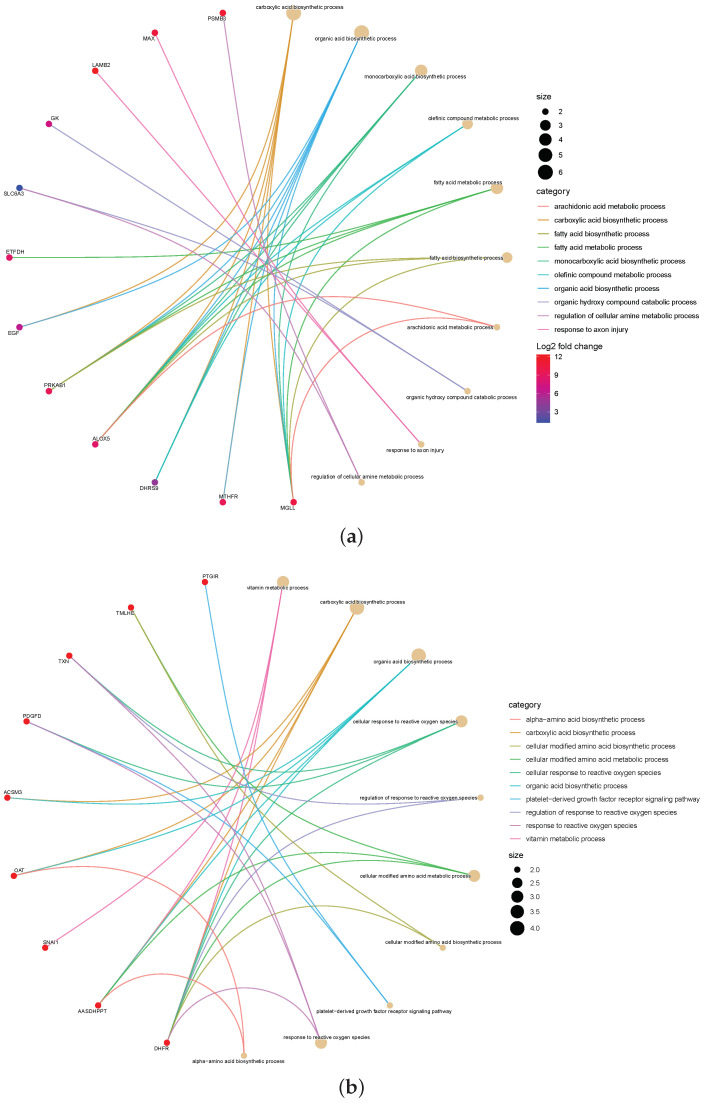

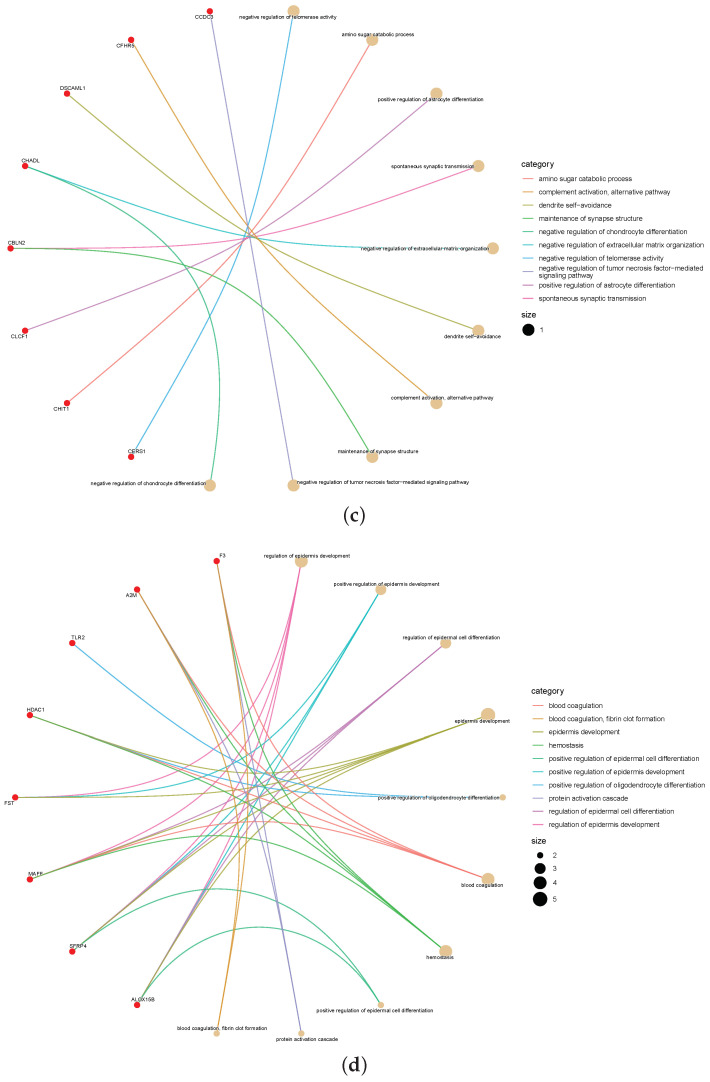
The gene–concept network depicts the linkages of genes and biological concepts as a network. (**a**) BRCA (breast cancer), (**b**) CESC (cervical carcinoma cancer), (**c**) COAD (colorectal cancer), and (**d**) PAAD (pancreas cancer).

Furthermore, to explore the significance of the omics signatures selected by the proposed method, we checked the interactions between the 20 top-ranked signatures from the mRNA by gene–concept network. Figure 5 shows the application of a gene–concept network to construct interaction networks between these mRNA of signatures. In each network analysis, the most important terms are listed, and the relevant genes are connected.

According to the color-mapping gene expression level, the connected terms are mapped with circles. Each gene is connected as a node, and each node is mapped to connected terms. The color scale of the related genes indicates the logFC in the highly expressed genes in the worst states. To find candidate genes as biomarkers for detecting HRA, each group was compared. For example, the mRNA signatures ETFDH, EGF, PRKAB1, ALOX5, DHRS9, MTHFR, and MGHH are in the maximum interaction network and are connected to other breast-cancer-related, frequently altered genes. In particular, ALOX5 plays a presumptive role in the breast cancer progression and patient prognosis [33].

In summary, we have used advanced machine learning to analyze disease genes in survival data, especially those already validated and deemed essential in oncology research. Our method and outcomes can be potentially assist clinicians or other medical researchers to properly explain the results of early-warning disease analysis.

## 4. Conclusions

In conclusion, our proposed DFSC algorithm can accurately improve the survival rate in cancer patient diagnosis. DFSC has been verified on four experimental datasets and has better prediction accuracy than the other four most advanced survival prediction models. Semi-supervised learning, an effective alternative method in the experimental process, can alleviate the challenge of over-fitting and improve the robustness of the model. Combining semi-supervised learning with a deep forest model can obtain better experimental results. In addition, DFSC can also be used to predict the survival rates of various high-dimensional and collinear diseases. By considering all categories at the same time in the gene selection stage, our proposed extension can identify genes, thereby allowing doctors to make more accurate computer-aided diagnoses.

The establishment of a model to understand the relationship between genomic features and patient survival is a challenge for the future. Advanced machine learning methods have become powerful tools for building an effective survival analysis model. We investigated current work to accurately identify genomic signatures associated with cancer patient survival to improve prognostic precision oncology.

## Figures and Tables

**Table 1:** Four tumor experimental datasets.

Dataset	Disease Type	No. of Samples	No. of Genes
BRCA	Breast	613	20,502
CESC	Cervical carcinoma	290	20,502
COAD	Colorectal	255	20,501
PAAD	Pancreas	176	20,502

**Table 2:** Details of the experimental dataset.

Dataset	Disease Type	Labeled Samples	Unlabeled Samples	Testing Samples	No. of Genes
BRCA	Breast	248	245	120	20,502
CESC	Cervical carcinoma	120	110	60	20,502
COAD	Colorectal	110	85	60	20,501
PAAD	Pancreas	65	61	50	20,502

**Table 3:** The top-20 genes found in the BRCA (breast cancer) dataset.

Gene Symbol	Stable Score	*p*-Value
A2M	0.99	<0.01
MGLL	0.98	<0.01
MTHFR	0.74	<0.01
PTGDR	0.79	<0.01
IL23A	0.79	<0.01
PSMB3	0.95	<0.01
HTR1F	0.97	<0.01
DHRS9	0.90	<0.01
MAGED1	0.99	<0.01
SLC6A3	0.84	<0.01
MED8	0.96	<0.01
LAMB2	0.91	<0.01
GK	0.91	<0.01
ALOX5	0.89	<0.01
ETFDH	0.90	<0.01
PKMYT1	0.93	<0.01
PRKAB1	0.91	<0.01
MAX	0.95	<0.01
EGF	0.97	<0.01
SLBP	0.97	<0.01

**Table 4:** The top-20 genes found in the CESC (cervical carcinoma cancer) dataset.

Gene Symbol	Stable Score	*p*-Value
TBCD	0.86	<0.01
CDC7	0.87	<0.01
ALDH3A2	0.96	<0.01
OAT	0.96	<0.01
FTL	0.94	<0.01
TRIP12	0.97	<0.01
NUP62	0.97	<0.01
DHFR	0.91	<0.01
AASDHPPT	0.90	<0.01
MAN1B1	0.89	<0.01
SNAI1	0.94	<0.01
ACSM3	0.84	<0.01
TMLHE	0.89	<0.01
PDGFD	0.81	<0.01
IFIT2	0.83	<0.01
GOSR1	0.79	<0.01
CPA3	0.78	<0.01
PTGIR	0.84	<0.01
NTRK2	0.83	<0.01
TXN	0.85	<0.01

**Table 5:** The top-20 genes found in the COAD (colorectal cancer) dataset.

Gene Symbol	Stable Score	*p*-Value
CBLN2	1.00	<0.01
CDC40	0.88	<0.01
CSMD3	0.88	<0.01
CHADL	0.73	<0.01
BOD1L1	0.80	<0.01
BRAP	0.86	<0.01
CLEC6A	0.82	<0.01
CCDC3	0.86	<0.01
C21orf58	0.91	<0.01
CFHR5	0.83	<0.01
C5orf58	0.84	<0.01
CCDC62	0.85	<0.01
CACNA2D3	0.78	<0.01
CERS1	0.87	<0.01
DSCAML1	0.83	<0.01
CLCF1	0.84	<0.01
COL10A1	0.82	<0.01
CHIT1	0.88	<0.01
CABYR	0.77	<0.01
CCDC148	0.81	<0.01

**Table 6:** The top-20 genes found in the PAAD (pancreas cancer) dataset.

Gene Symbol	Stable Score	*p*-Value
A2M	0.90	<0.01
GZMA	0.92	<0.01
SEPHS2	0.95	<0.01
ANAPC10	0.96	<0.01
ALOX15B	0.89	<0.01
TNNI1	0.92	<0.01
CDIPT	0.89	<0.01
MCCC1	0.89	<0.01
ZNF3	0.89	<0.01
F3	0.86	<0.01
TLR2	0.85	<0.01
SFRP4	0.84	<0.01
CTNNBL1	0.82	<0.01
GNRH1	0.86	<0.01
MAFF	0.81	<0.01
ARHGEF3	0.80	<0.01
HDAC1	0.82	<0.01
FST	0.83	<0.01
B3GALT2	0.82	<0.01
CA2	0.82	<0.01

## Data Availability

The data presented in this study are openly available in TCGA data portal at https://portal.gdc.cancer.gov/.

## References

[1] Wang Q., Zhou Y. (2022). FedSPL: Federated self-paced learning for privacy-preserving disease diagnosis. Brief. Bioinform..

[2] Yang X., Kui L., Tang M., Li D., Wei K., Chen W., Miao J., Dong Y. (2020). High-throughput transcriptome profiling in drug and biomarker discovery. Front. Genet..

[3] Katzman J.L., Shaham U., Cloninger A., Bates J., Jiang T., Kluger Y. (2018). DeepSurv: Personalized treatment recommender system using a Cox proportional hazards deep neural network. BMC Med. Res. Methodol..

[4] Lánczky A., Győrffy B. (2021). Web-based survival analysis tool tailored for medical research (KMplot): Development and implementation. J. Med. Internet Res..

[5] Panda S.K., Cheong H., Tun T.A., Devella S.K., Senthil V., Krishnadas R., Buist M.L., Perera S., Cheng C.Y., Aung T. (2022). Describing the structural phenotype of the glaucomatous optic nerve head using artificial intelligence. Am. J. Ophthalmol..

[6] Chen X., Ishwaran H. (2012). Random forests for genomic data analysis. Genomics.

[7] Breslow N.E. (1984). Extra-Poisson variation in log-linear models. J. R. Stat. Soc. Ser. C (Appl. Stat.).

[8] Ma B., Yan G., Chai B., Hou X. (2021). XGBLC: An improved survival prediction model based on XGBoost. Bioinformatics.

[9] Lee C., Zame W., Yoon J., Van Der Schaar M. Deephit: A deep learning approach to survival analysis with competing risks. Proceedings of the AAAI Conference on Artificial Intelligence.

[10] Van Belle V., Pelckmans K., Van Huffel S., Suykens J.A. (2011). Improved performance on high-dimensional survival data by application of Survival-SVM. Bioinformatics.

[11] Huang Z., Zhan X., Xiang S., Johnson T.S., Helm B., Yu C.Y., Zhang J., Salama P., Rizkalla M., Han Z. (2019). SALMON: Survival analysis learning with multi-omics neural networks on breast cancer. Front. Genet..

[12] Huang Z., Johnson T.S., Han Z., Helm B., Cao S., Zhang C., Salama P., Rizkalla M., Yu C.Y., Cheng J. (2020). Deep learning-based cancer survival prognosis from RNA-seq data: Approaches and evaluations. BMC Med. Genom..

[13] Ishwaran H., Gerds T.A., Kogalur U.B., Moore R.D., Gange S.J., Lau B.M. (2014). Random survival forests for competing risks. Biostatistics.

[14] Zhou Z., Feng J. (2019). Deep Forest. Nat. Sci. Rev..

[15] Utkin L., Konstantinov A., Meldo A., Sokolova V., Coolen F. (2021). The Deep Survival Forest and Elastic-Net-Cox Cascade Models as Extensions of the Deep Forest. Proceedings of the International Scientific Conference on Telecommunications, Computing and Control.

[16] Chen Y., Li Y., Narayan R., Subramanian A., Xie X. (2016). Gene expression inference with deep learning. Bioinformatics.

[17] Zhang Y., Li M., Ji Z., Fan W., Yuan S., Liu Q., Chen Q. (2021). Twin self-supervision based semi-supervised learning (TS-SSL): Retinal anomaly classification in SD-OCT images. Neurocomputing.

[18] Liu F., Tian Y., Cordeiro F.R., Belagiannis V., Reid I., Carneiro G. (2021). Self-supervised mean teacher for semi-supervised chest X-ray classification. Proceedings of the International Workshop on Machine Learning in Medical Imaging.

[19] Song L., Feng Z., Yang S., Zhang X., Jiao L. (2022). Self-Supervised Assisted Semi-Supervised Residual Network for Hyperspectral Image Classification. Remote. Sens..

[20] Wang Q., Zhou Y., Ding W., Zhang Z., Muhammad K., Cao Z. (2020). Random Forest with Self-paced Bootstrap Learning in Lung Cancer Prognosis. ACM Trans. Multimed. Comput. Commun. Appl. (TOMM).

[21] Sun L., Mo Z., Yan F., Xia L., Shan F., Ding Z., Song B., Gao W., Shao W., Shi F. (2020). Adaptive feature selection guided deep forest for COVID-19 classification with chest ct. IEEE J. Biomed. Health Inform..

[22] Zhu Q., Pan M., Liu L., Li B., He T., Jiang X., Hu X. An Ensemble Feature Selection Method Based on Deep Forest for Microbiome-Wide Association Studies. Proceedings of the 2018 IEEE International Conference on Bioinformatics and Biomedicine (BIBM).

[23] Yu B., Chen C., Wang X., Yu Z., Ma A., Liu B. (2021). Prediction of protein–protein interactions based on elastic net and deep forest. Expert Syst. Appl..

[24] Xin B., Hu L., Wang Y., Gao W. Stable feature selection from brain sMRI. Proceedings of the Twenty-Ninth AAAI Conference on Artificial Intelligence.

[25] Liu W., Lin H., Huang L., Peng L., Tang T., Zhao Q., Yang L. (2022). Identification of miRNA–disease associations via deep forest ensemble learning based on autoencoder. Brief. Bioinform..

[26] Kantidakis G., Putter H., Lancia C., Boer J.d., Braat A.E., Fiocco M. (2020). Survival prediction models since liver transplantation-comparisons between Cox models and machine learning techniques. BMC Med. Res. Methodol..

[27] Heller G. (2020). The added value of new covariates to the brier score in cox survival models. Lifetime Data Anal..

[28] Mordelet F., Horton J.R., Hartemink A.J., Engelhardt B.E., Gordân R. (2013). Stability selection for regression-based models of transcription factor–DNA binding specificity. Bioinformatics.

[29] Jawale R.M., Williams K., Lee M., Yang H.H., Figueroa J., Sherman M., Otis C.N., Arcaro K. (2014). Tamoxifen-resistant breast cancer: DNA methylation and expression of MAGED1. Cancer Res..

[30] Wang L., Zhang P., Molkentine D.P., Chen C., Molkentine J.M., Piao H., Raju U., Zhang J., Valdecanas D.R., Tailor R.C. (2017). TRIP12 as a mediator of human papillomavirus/p16-related radiation enhancement effects. Oncogene.

[31] Haddick P., Irene T., Elizabeth L., Gabriel Q., Wranik B.J., Ramani S.R., Jean-Philippe S., Marc T.L., Gonzalez L.C., Brian K. (2014). Defining the Ligand Specificity of the Deleted in Colorectal Cancer (DCC) Receptor. PLoS ONE.

[32] Johnston J.R., Chase P.B., Pinto J.R. (2018). Troponin through the looking-glass: Emerging roles beyond regulation of striated muscle contraction. Oncotarget.

[33] Malanchi I. (2015). Neutrophils Promote ALOX5-Dependent Breast Cancer Lung Metastasis. Cancer Discov..

